# Advances in extracorporeal membrane oxygenation: investing in training of multidisciplinary teams and developing health care infrastructure: In Reply to: Healthcare professionals’ views on training, standards, and resources for extracorporeal membrane oxygenation

**DOI:** 10.3325/cmj.2026.67.133

**Published:** 2026-04

**Authors:** Bekzhan A. Permenov, Burhan Fatih Kocyigit

**Affiliations:** 1 Department of Cardiac Surgery, Anesthesiology, and Intensive Care, Heart Center Shymkent, Shymkent, Kazakhstan; 2 Department of Social Health Insurance and Public Health, South Kazakhstan Medical Academy, Shymkent, Kazakhstan; 3 Department of Internal Medicine, Khoja Akhmet Yassawi International Kazakh-Turkish University, Turkistan, Kazakhstan; 4 Department of Physical Medicine and Rehabilitation, University of Health Sciences, Adana City Research and Training Hospital, Adana, Turkey

We thank Durmaz and Durmaz (1) for the opportunity to discuss the implications of our latest survey on extracorporeal membrane oxygenation (ECMO) (2) and clarify a few related points. This letter aims to elucidate some methodological and conceptual aspects of our survey. It is drafted in line with the LETTERS recommendations of the EQUATOR network (3).

The comment on our study suggests that our discussion should be more detailed. Cross-sectional surveys capture perceived realities at a certain point in time. Importantly, our self-administered questionnaire was structured based on the Extracorporeal Life Support Organization (ELSO) guidelines (https://www.elso.org/ecmo-resources/elso-ecmo-guidelines.aspx). The published report followed the recommendations on questionnaire designs, reporting standards, and dissemination strategies outlined in the seminal work by Gasparyan and Zimba (4).

Aligning our ECMO questionnaire with internationally recognized standards enhances the instrument's content validity and ensures that the evaluated domains – training availability, certification awareness, resource barriers, and multidisciplinary team issues – accurately reflect the constructs identified by the ELSO guidelines as essential determinants of ECMO program quality. The perceptions of health care professionals regarding these domains are of substantial practical significance, as attitudinal and knowledge-based barriers are major contributors to implementation gaps, regardless of objective institutional assessments. Future research that integrates self-reported data with objective institutional measurements could yield a more comprehensive understanding, and researchers in well-resourced environments are encouraged to pursue such methodologies.

We respectfully acknowledge that concerns about respondents from numerous countries were explicitly addressed in our index publication (2). In the Discussion section, we stated that “a relatively small sample size and convenience sampling restrict the generalizability of the findings,” and that “respondents were predominantly from Kazakhstan”. Additionally, the conclusion explicitly states that “our results primarily reflect practices in Kazakhstan and should be interpreted in light of the study’s restricted geographical coverage.” Geographical and sampling limitations were communicated transparently in accordance with established standards for survey-based studies. The observed geographic concentration stems from the limitations of convenience sampling through social media, a widely adopted approach for online surveys. Nonetheless, our findings possess significant policy relevance for a range of health care settings beyond Kazakhstan. In fact, recent evidence further emphasizes the necessity for systematic nursing training in ECMO, demonstrating that outcomes in complex ECMO cases, such as those involving intracranial hemorrhage, are closely linked to the coordinated competence of the entire multidisciplinary team (5).

Admittedly, there is a gap between individual specialist knowledge and system-level capacity. Our survey captures both aspects: questions on personal familiarity with ELSO guidelines and ECMO definitions assess individual competence, while items on departmental ECMO availability, annual case volumes, and access to training programs assess institutional readiness. High ECMO costs and limited device availability are systemic barriers that individual training alone cannot overcome without investment in health care infrastructure and reimbursement policy. Disseminating accurate, high-quality ECMO information, including through popular digital platforms, remains a challenge at both individual and system levels (6). Future research should more clearly separate these levels of analysis.

In conclusion, our survey report contributes to understanding the constraints of ECMO implementation in resource-limited settings. We remain committed to advancing this field through multidisciplinary research.
